# A Personalized Approach Using an N-of-1 Trial of Virtual Reality Hypnosis for Persistent Dyspnea

**DOI:** 10.1089/jmxr.2024.0029

**Published:** 2024-11-14

**Authors:** Nils Siegenthaler, Hugo Bothorel, Oliver Alan Kannape, Capucine Morelot-Panzini, Dan Adler

**Affiliations:** 1 Intensive care Unit, Hôpital de la Tour, Geneva, Switzerland.; 2 Faculty of Medicine, University of Geneva, Geneva, Switzerland.; 3 Research Department, Hôpital de la Tour, Geneva, Switzerland.; 4 Virtual Medicine Centre, HUG-NeuroCentre, Department of Clinical Neurosciences, Geneva University Hospitals, Geneva, Switzerland.; 5 Sorbonne Université, INSERM, UMRS 1158, Neurophysiologie Respiratoire Expérimentale et Clinique, Paris, France.; 6 Division of Pulmonary Diseases, Hôpital de la Tour, Geneva, Switzerland.

**Keywords:** dyspnea, virtual reality hypnosis, N-of-1 trial

## Abstract

Persistent dyspnea is associated with major suffering in chronic obstructive pulmonary disease (COPD) patients. Addressing this clinical challenge requires personalized approaches that account for individual variability in treatment response. N-of-1 trials offer a promising method to evaluate interventions tailored to individual patients. This study utilizes N-of-1 trials to assess the effect of virtual reality hypnosis (VRH) therapy on persistent dyspnea to inform personalized therapeutic decisions. Two COPD patients with persistent dyspnea participated in N-of-1 trials, each consisting of four counterbalanced periods of exposure to different virtual reality conditions (period A: VRH; period B: a distractive, pleasant drone ride) over 2 consecutive days. Through patient-generated problem-specific questionnaires, outcomes were evaluated on a 6-point Likert scale. Results showed significant reductions in dyspnea intensity and improved well-being during VRH for one patient, while the other reported improved well-being without significant changes in dyspnea. Our report illustrates the potential of N-of-1 trials in evaluating personalized treatment efficacy, highlighting the importance of customized approaches when conventional treatments have been exhausted. Incorporating N-of-1 trials of alternative treatments in the dyspnea clinic may enhance patient outcomes and satisfaction by tailoring interventions to individual patient needs.

## Introduction

Physicians implicitly combine scientific evidence, clinical judgment, and patients’ preferences to inform therapeutic decisions. However, evidence derived from conventional randomized controlled trials only generates an average treatment effect in a specific study population. It does not and cannot predict if a given intervention will be effective and appropriate for one individual. Conversely, personalized medicine emphasizes individualized treatment based on a demonstrated positive and meaningful outcome for one individual. N-of-1 trials are multiple crossover trials in single individuals. They are very robust to high heterogeneity of effects between patients, non-ideal conditions, differing co-morbidities, and other potentially confounding factors because the unit of analysis is a single patient.^1^

Persistent dyspnea refers to the distressing awareness of respiratory activity experienced once all pathophysiological treatments of cardiorespiratory dysfunction have been optimized. This experience is associated with fear, anxiety, and a disabling feeling of loss of control.^2,3^ Persistent dyspnea activates shared neural networks with pain processing and bodily self-consciousness, including the insula, the dorsal anterior cingulate cortex, the amygdala, and the thalamus.^4^ Furthermore, experiencing a difficult, traumatic event can lead to emotional memorization of the experience. This in turn reinforces the traumatic aspect of new episodes and contributes to anxious anticipation of their reappearance. It is therefore relevant to target these brain processes and influence the perception of the lived experience to contribute holistically to the management of persistent dyspnea when all pathophysiological treatments have been exhausted.^5^ In the field of respiratory neurophysiology, virtual reality (VR)-based therapeutics improve the feeling of breathing control (sense of agency),^6^ reduce the negative emotional state associated with experimental dyspnea,^7^ modify the breathing pattern,^8^ and have also been associated with alleviation of dyspnea in patients recovering from coronavirus-19 (COVID-19) pneumonia.^9^

Among the available VR hypnosis (VRH) platforms, HypnoVR (HypnoVR, Strasbourg, France) offers a solution that combines visual immersion with scripted hypnotic voice-tracks. Their solution has been certified as a medical device and has been studied in various medical situations. The hardware setup includes a head-mounted display (Pico G3, ByteDance, Beijing, China), a pair of noise-cancelling Bluetooth headphones (Eonoheadphone 1, Eono), and a tablet (M9 Tab, Lenovo Group Limited, Beijing, China). Patients can select from various virtual environments and background music, while the therapist can further choose the hypnotic script and the duration via the application on the tablet.

Here, we report the results of a simple method to test the effect of VRH in persistent dyspnea. We hypothesized that N-of-1 single trials of VRH therapy in persistent dyspnea could uniquely inform therapeutic decisions in individual patients, thereby closing a major gap between evidence and clinical practice. Therefore, the aim of this N-of-1 single trial was to determine the utility of VRH therapy on two outcomes determined by patients themselves.

## Case Presentation

**FIG. 1.**
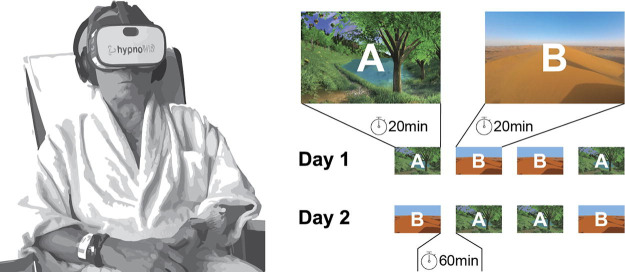
Experimental setup (left) and experimental design (right) for the VRH based N-of-1 trial in persistent dyspnea. Each period (A: VRH and B: drone ride) is terminated with the recording of outcomes and a 1-h washout time before next experimental period. A second sequence of 4 periods is repeated on day 2. VRH, virtual reality hypnosis.

We used a repeated sequence of four counterbalanced periods of exposure to two conditions (A and B) on 2 consecutive days, as shown in Figure 1. Period A corresponded to a 20-min exposure to VRH by coupling 3D immersion in dynamic scenery (walking in a forest) with a standardized hypnotic script. Period B corresponded to a 20-min exposure to a pleasant drone flight across a desert, at slow speeds, but without any hypnotic script (distraction). The first 2 min in period A corresponded to an induction period, whereas the last 2 min were intended to lead the patient back into the present situation. A washout period of 1 h was used between each exposure. The patients had never been exposed to VRH before and had no counterindication to VRH. They were in stable respiratory and cardiac conditions and denied any neurological or psychiatric diseases. The patients and the nurses in charge of delivering therapy and data acquisition were not blinded to the experimental periods. The biostatistician and the physician were blinded to treatment allocation for data analysis. Since case reports do not require approval from an ethics committee in the Canton of Geneva, we waived this requirement. Written consent was obtained from both patients prior to testing VRH.

The primary and secondary outcomes were determined by the patients themselves and measured after each exposure to VRH or to distraction using the “Measure Yourself Medical Outcome Profile” (MYMOP).^10^ This patient-generated problem-specific questionnaire uses standardized wording and response options openly discussed with the patients to align with their main concerns. A 6-point Likert scale is used to assess two personalized outcomes ranging from 1 (as good as it can be) to 6 (as bad as it can be). The general feeling of wellbeing was also assessed after each period of exposure using the same Likert scale.

Descriptive statistics were used to summarize the data. Continuous variables were reported as mean ± standard deviation (min–max). The normality of continuous variable distributions was assessed by the Shapiro–Wilk test. The significance of outcomes differences for each patient between the repeated intervention (A) and sham (B) periods was assessed using a one-sided Wilcoxon-signed rank test. The analyses were performed using R (version 3.6.2, R Foundation for Statistical Computing, Vienna, Austria), and *p* values of <0.05 were considered significant.

**FIG. 2.**
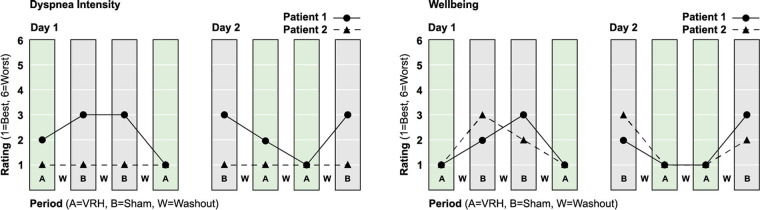
Left: Dyspnea intensity ratings by patients 1 and 2 after periods of VRH (A) and sham exposure (B). Patient 1 reported less intense dyspnea after a period of VRH compared with the sham condition (B) whereas no effect of VRH or sham exposure was observed in patient 2. Right: Wellbeing ratings by patients 1 and 2 after periods of VRH (A) and sham exposure (B). Both patients reported a greater feeling of wellbeing after a period of VRH (A) than after a period of simple distraction (B). VRH, virtual reality hypnosis.

The first patient invited to participate in this N-of-1 trial was much involved in his own care and highly motivated to test non-pharmacological therapies targeting brain mechanisms for persistent dyspnea. He was a 75-year-old male with GOLD 4E chronic obstructive pulmonary disease (COPD) struggling with persistent dyspnea during an acute episode of severe exacerbation of COPD. He suffered from persistent dyspnea despite treatment with oxygen therapy, maximal inhaled bronchodilators, systemic corticosteroids, diuretics, and broad-spectrum antibiotics. He has not received morphine because of non-acidotic minimal hypercapnia. Multiple exposures to period A had a positive impact on intensity of dyspnea (the first outcome generated by the patient) compared with period B (1.5 ± 0.6 vs. 3.0 ± 0.0; *p* = 0.032; Supplementary Table S1), as shown in Figure 2. No effect was observed between the periods A and B on anger (the second outcome generated by the patient) (1.0 ± 0.0 vs. 1.0 ± 0.0). Period A was systematically associated with an improved feeling of wellbeing compared with period B (1.0 ± 0.0 vs. 2.5 ± 0.6, *p* = 0.032). The patient did not report any feeling of discomfort or strangeness while using VRH. He indicated that he would like to continue to use VRH during his recovery in the hospital but was not ready to use VRH at home for persistent dyspnea.

The second patient was a 73-year-old female known for GOLD 4E COPD and lung volume reduction surgery presenting for persistent dyspnea to our outpatient clinic. Despite receiving optimal dual bronchodilation and maintenance pulmonary rehabilitation, she continued to experience distressing awareness of respiratory activity, leading to fear, anxiety, and a loss of control. Multiple exposures to period A had neither any effect on intensity of dyspnea (first outcome generated by the patient) nor on anxiety (the second outcome generated by the patient) compared with multiple exposures to period B (Supplementary Table S1). Nevertheless, period A was systematically associated with an improved feeling of wellbeing compared with period B (1.0 ± 0.0 vs. 2.5 ± 0.6, *p* = 0.032) (shown in Fig. 2). The patient did not report any feeling of discomfort or strangeness while using VRH. She enjoyed the intervention and would accept to use VRH during supervised sessions in pulmonary rehabilitation.

## Discussion

This N-of-1 trial of VRH therapy for persistent dyspnea sheds light on the potential effectiveness of this intervention for individual patients. This trial was also specifically designed to test a simple, robust, and replicable method aimed at helping clinicians determine the utility of an additional non-pharmacological approach, such as VRH therapy, for the management of persistent dyspnea.

The first illustrative patient experienced a significant reduction in the intensity of dyspnea when exposed to VRH therapy compared with a drone ride with no hypnotic script. Throughout the course of the VRH therapy, the patient reported a significant reduction in the intensity of dyspnea. However, VRH had no effect on dyspnea-related anger, the second important self-generated outcome. The patient also reported an overall feeling of well-being during VRH exposure compared with the drone ride. On the other hand, the second patient, a 73-year-old female with severe COPD and a history of lung volume reduction surgery, neither experienced any significant changes in dyspnea intensity (the first self-generated outcome) nor any modification of anxiety (the second self-generated outcome) when exposed to VRH therapy. As with the first patient, she reported an improved feeling of well-being during the VRH sessions compared with the pleasant drone ride.

The divergent outcomes underscore the significance of individual variability in treatment response while also highlighting the disparity between the ideal controlled research environment, where treatment effects are tested on groups of patients, and the complex, real-world conditions encountered at the bedside when decisions must be made for a single patient. Moreover, there is a long chain of events between a dyspnogenic stimulus and what the patient reports. These include perceptual/sensory processes and cognitive/affective processes, with many sources of modulation (i.e., psychological, cultural, and social), making dyspnea a multidimensional and highly subjective experience. Therefore, implementing simple methods such as N-of-1 trials into clinical practice to assess treatment efficacy for individual patients represents a crucial step towards personalized medicine. It involves considering the unique preferences, goals, and responses of each patient when making therapeutic decisions.

The positive impact of VRH therapy on dyspnea and well-being in the first patient supports previous research suggesting the potential benefits of VR-based therapeutics in respiratory conditions. VR has been shown to improve the sense of breathing control, negative emotions associated with dyspnea, and modify breathing patterns in experimental and clinical settings.^6–8^ The activation of shared neural networks involved in pain processing and bodily self-consciousness further supports the rationale for targeting the brain in interventions for persistent dyspnea.^4^

## Conclusion 

In conclusion, our report illustrates a simple method to demonstrate the potential of VRH therapy as a personalized treatment option for persistent dyspnea in individual patients. While our results indicate positive effects on dyspnea and well-being in one patient, they also highlight the importance of using a robust method to test the effects of VRH. By incorporating N-of-1 trials in personalized medicine principles, healthcare professionals can close the gap between evidence-based medicine and clinical practice, ultimately improving patient outcomes and satisfaction.

## Data Availability

The data that support this case report are available from the corresponding author on reasonable request.

## Abbreviations Used

COPD: Chronic obstructive pulmonary disease
COVID-19: Coronavirus-19
GOLD: Global Initiative for Chronic Obstructive Lung Disease
MYMOP: Measure yourself medical outcome profile
VR: Virtual reality
VRH: Virtual reality hypnosis

## References

[1] Lillie EO, Patay B, Diamant J, et al. The n-of-1 clinical trial: The ultimate strategy for individualizing medicine? Per Med, 2011; 8(2):161–173. 21695041 10.2217/pme.11.7 PMC3118090

[2] Morélot-Panzini C, Adler D, Aguilaniu B, et al. dyspnoea working group of the Société de Pneumologie de Langue Française. Breathlessness despite optimal pathophysiological treatment: On the relevance of being chronic. Eur Respir J, 2017; 50(3). 10.1183/13993003.01159-2017 28954773

[3] Parshall MB, Schwartzstein RM, Adams L, et al. American Thoracic Society Committee on Dyspnea. An official American thoracic society statement: Update on the mechanisms, assessment, and management of dyspnea. Am J Respir Crit Care Med, 2012; 185(4):435–452. 22336677 10.1164/rccm.201111-2042ST PMC5448624

[4] Betka S, Adler D, Similowski T, et al. Breathing control, brain, and bodily self-consciousness: Toward immersive digiceuticals to alleviate respiratory suffering. Biol Psychol, 2022; 171:108329. 35452780 10.1016/j.biopsycho.2022.108329

[5] Similowski T . Treat the lungs, fool the brain and appease the mind: Towards holistic care of patients who suffer from chronic respiratory diseases. Eur Respir J, 2018; 51(2). 10.1183/13993003.00316-2018 29496732

[6] Adler D, Herbelin B, Similowski T, et al. Reprint of “Breathing and sense of self: Visuo-respiratory conflicts alter body self-consciousness. Respir Physiol Neurobiol, 2014; 204:131–137. 25266397 10.1016/j.resp.2014.09.019

[7] Allard E, Canzoneri E, Adler D, et al. Interferences between breathing, experimental dyspnoea and bodily self-consciousness. Sci Rep, 2017; 7(1):9990. 28855723 10.1038/s41598-017-11045-y PMC5577140

[8] Betka S, Canzoneri E, Adler D, et al. Mechanisms of the breathing contribution to bodily self-consciousness in healthy humans: Lessons from machine-assisted breathing? Psychophysiology, 2020; 57(8):e13564. 32162704 10.1111/psyp.13564 PMC7507190

[9] Betka S, Kannape OA, Fasola J, et al. Virtual reality intervention alleviates dyspnoea in patients recovering from COVID-19 pneumonia. ERJ Open Res, 2023; 9(6). 10.1183/23120541.00570-2022 PMC10658613 38020572

[10] Paterson C . Measuring outcomes in primary care: A patient generated measure, MYMOP, compared with the SF-36 health survey. BMJ, 1996; 312(7037):1016–1020. 8616351 10.1136/bmj.312.7037.1016 PMC2350813

